# The role of the livestock auction mart in promoting help-seeking behavior change among farmers in the UK

**DOI:** 10.1186/s12889-022-13958-4

**Published:** 2022-08-20

**Authors:** Caroline Nye, Michael Winter, Matt Lobley

**Affiliations:** Centre for Rural Policy Research (CRPR), University of Exeter, Lazenby House, Prince of Wales Road, Exeter, EX4 4PJ UK

**Keywords:** Livestock auction mart, Market, Help-seeking, Mental health, Suicide, Farmers

## Abstract

Certain physical and mental health issues are particularly prevalent in farming occupations, yet frequently, farmers, particularly males, are resistant to seeking help from primary care practitioners. A qualitative approach examined the perspective of stakeholders at livestock auction marts to identify the determinants for, or barriers to, seeking help, perceptions regarding basing primary care services on-site at livestock auction marts, and the role of a site-based approach, i.e. placing primary healthcare services within a traditional farmers’ meeting place, in facilitating changes in help-seeking beliefs and behaviors. Findings support previous studies regarding barriers to seeking help, but demonstrate that by deconstructing these barriers through specifically designed workplace/site-oriented support services, more positive behaviors are facilitated. The study highlights how collaboration between livestock auction marts and primary healthcare services allows access to a hard-to-reach demographic in terms of healthcare, and illustrates how such socially integrative opportunities can contribute to the improvement of the mental and physical health and wellbeing of the agricultural community.

## Introduction

Individuals working in farming in the UK are particularly susceptible to a number of health conditions and workplace injuries [1]. This pattern is mirrored by farming communities in regions such as Australasia, North America and some areas of Europe [2–4]. Agriculture, forestry and fishing occupations in the UK have twice the rate of workplace non-fatal injury than other main industry sectors, although rates are likely to be even higher as incidents are known to be substantially underreported [5]. Around half of all work-related ill health cases in farming are musculoskeletal disorders, while respiratory illness, skin disorders and zoonotic infections are also common [5, 6]. Many farmers are also prone to poor diet and lifestyle choices, such as smoking or alcohol consumption, and are therefore vulnerable to the secondary health implications which might stem from these [7]. In addition, poor mental health is common among people working in farming, with 84% of farmers under the age of 40 considering mental health issues to be the biggest danger facing agriculture [8]. Mental health issues in farming include stress, depression, anxiety or PTSD [9], and often stem from challenges arising from the agricultural industry, such as changes in policy or regulatory pressures, or from issues related to loneliness or rural isolation. In spite of the prevalence of health issues within the industry, help-seeking by farmers from health professionals remains relatively low, particularly for men [10–12]. Male farmers are recognized as being especially hard-to-reach with regards to help-seeking, health promotion interventions, and preventive health behaviors [7]. Hurley et al. [13] describe the term ‘hard-to-reach’ as referring to farmers who are ‘difficult to contact or engage with and therefore often omitted from research, policy and underserved by extension services’ [13] (p.748). Not only is this likely to lead to poor prognoses of physical health issues due to a delay in treatment, but the impact of untreated physical or mental health symptoms can increase stress and negatively impact upon farm productivity, farm income, family life, and animal health and welfare [7, 11, 14].

According to Jackson et al. [15], ‘help-seeking’ differs from ‘service utilization’ in that it encompasses ‘a range of indicators including attitudes to seeking help, planned behavior, and consultation with friends, help lines, the internet or professionals’ [15] (p. 148). This article explores approaches to help-seeking using the basic components of the Health Belief Model (HBM) [16]. The value expectancy theory HBM was selected as the most appropriate theoretical model, over and above other models such as the Theory of Planned Behavior (TPB) [17] and the Theory of Reasoned Action (TRA) [18] due to its emphasis on understanding why individuals do not participate in health-related behaviors, as opposed to examining behavioral intent more generally. Originating from a group of social psychologists in the early 1950s, the HBM was developed to understand ‘the widespread failure of people to accept disease preventatives or screening tests for the early detection of asymptomatic disease’ [16] (p.328). It orientates around four dimensions of perception regarding health: the *perceived susceptibility* of an individual to a certain condition, the *perceived severity* of an illness in terms of its physical and social consequences, the *perceived benefits* of seeking help and receiving treatment, and the *perceived barriers* associated with engaging in a particular health action. While all dimensions of the model are considered in this study, emphasis is given to the perceived barriers and benefits of help-seeking, as these two dimensions are considered to drive the choice between seeking and not seeking help. Perceived barriers is also, according to Janz and Becker [19] ‘the most powerful HBM dimension’ [19] (p. 37). Perceived severity is more challenging to investigate due to difficulties respondents might find in ‘conceptualizing this dimension’ [19] (p. 36). Demographic and psychosocial factors, such as age, personality, and education, as well as cues to action, also play a lesser role in the importance of the HBM, and while these will be considered in our analysis, it will be done so under one of the main component dimensions.

Multiple studies have highlighted a number of determinants and barriers driving decision-making processes around help-seeking, with a particular emphasis on men. Confidentiality, acceptability, pride, geographical isolation, lack of service awareness [20], perceived stigma attached to help-seeking behaviors [21], competing demands on time and resources, sense of responsibility towards farm production, fear of ridicule [22] and the threat to integral identity [23]) are just a few of the determinants identified. With regards to rural workers, the belief is that hegemonic masculinity in the rural domain perpetuates the embodiment of traditional perceptions that men are tough in the face of adversity, which subsequently influences behaviors towards help-seeking for health issues [10, 24]. The benefits of inhabiting a role of dominant masculinity within the rural domain has allowed some men positions of influence and power, and of ‘pre-eminence in public life and ownership and control of most of the resources of agriculture’ [10] (p. 136). However, the stoic behavior which has become inherent in the playing out of such roles is believed to lead to resistance to seeking help where needed, potentially resulting in a negative outcome. Identity is tightly tied to the role of being a farmer in the rural context, and exhibiting weakness by seeking help presents a threat to the identity of a male farmer which supersedes the importance of his own health and wellbeing [10].

De Visser et al. [25] consider masculinity in terms of ‘credit’ or ‘capital’, something which can be accrued, and possibly traded, within certain settings. This borrows from Bourdieu’s theory of capital [26], particularly symbolic capital– the form of capital which legitimizes social, cultural and economic capital in the form of recognition or prestige—and expresses the idea that masculine capital ‘can be used to allow or compensate for nonmasculine behavior’ [27] (p. 6). They suggest that in general, masculinity and femininity are not necessarily irreconcilable, but that ‘within particular behavioral domains masculinity and femininity tend to be seen as oppositional’ [27] (p. 12). This might explain how the role of women in recognizing the need for male farmers to seek help often proves the crucial impetus for converting passive behaviors to more active, help-seeking behaviors [10, 11]. Robertson [28] identifies the danger of relying too much on women to encourage help-seeking by males, suggesting that it can reinforce normative behavior and identities of masculine hegemony by allowing men to claim they had been ‘pressured into attending’ rather than choosing to seek help of their own free will [28] (p. 113).

Within rural communities, livestock auction marts have provided the ideal scenario for the accrual of masculine capital. Auction marts have been described as sites which have, traditionally, been ‘physically, metaphorically, culturally and architecturally expressive of masculinity’ [29] (p. 71). Superficially, this suggests that the deconstruction of behavioral norms related to help-seeking might not be best attempted within such a domain. Roy et al. [20], however, suggest behavioral norms are changing, with pride less likely to be an obstacle to help-seeking among younger farmers. They suggest that ‘rural masculine practices are fluid, rather than static’ [20] (p. 472) and that new beliefs and behaviors can therefore be encouraged through proactive measures to address the social determinants of help-seeking among farmers.

The livestock auction mart has long played a significant social role within agricultural communities, acting as a space within which a network of individuals are joined by ‘shared backgrounds, shared information, shared rituals, shared ideas about masculinity, and ultimately, shared identity’ [29] (p. 95). Yet, few empirical studies exist contemplating their role beyond the buying and selling of animals. The structure of livestock auction marts, and the dynamics played out within them, will likely have perpetuated behavioral norms, especially those driving expressions of masculinity and stoicism, while at the same time acting as sites of informal support should an individual need to talk. However, the last 20 years have seen a gradual transition at some auction mart sites which not only challenge such entrenched norms, but encourage and facilitate new behaviors following recognition of the need for, and importance of, greater levels of help-seeking among the auction-going community. This is largely seen in the provision of services on-site, from purpose-built health clinics, to the presence of rural chaplains, to signposting mental health services. One study revealed that 18 per cent of surveyed marts offer some form of primary healthcare service on-site [30]. Such transformations within the mart space are in line with wider societal changes in the global north and mirror evidence that a greater number of rural men want to ‘align their perceptions of masculinities with healthier practices’ [31]. These transitions in attitudes towards health and wellbeing have been assisted through promotion by multiple campaigns, as well as by an increase in the number of support organizations available across the UK [32]. The media also contributes to shifts in perceptions of help-seeking among men [33]. While help-seeking among farmers has received some attention within academic literature, the increase of primary healthcare services provided on-site at livestock auction marts, and the role this has played in encouraging new behavioral norms among farmers, are scarcely examined [34, 35].

The late 1990s witnessed the beginnings of a change in strategy regarding rural health services. Gerrard [36] examined occupational health issues in agricultural communities, revealing that the majority of farmers in the study felt their needs were not being met, and highlighting that farmers as an occupational group were ‘vulnerable in terms of the lack of attention given to their health and occupational health needs’ [36] (p. 162). The physical and mental health of farmers continued to be a growing concern during the 1990s, following a period of hardship and economic decline in agriculture [37]. It was recognized that if the farmer would, or could, not go to the doctor, perhaps efficient service delivery required facilitation in the form of matching a needs-specific, ‘work-based’ service directly with the farmer. At the same time, it was acknowledged that social, cultural and in some cases, language, barriers required breaking down between primary healthcare providers and the farming community in a bid to encourage more positive help-seeking behaviors and make healthcare service provision more accessible [38]. Auction marts were identified early on as prime sites from which to offer this new, targeted provision, and in 1999 a nurse-practitioner-led farmers’ health project was initiated in the South Cumbria and North Lancashire area [34, 37]. Inspired by the nurse practitioner movement which began in North America in the 1970s, this initiative provided a service tailored to the needs of farmers, and was delivered from a mobile vehicle. One of their most significant findings revealed that 56 per cent of patients seen by the nurses were found to have significant health problems, but that only 24 per cent of those were managing the health problem with their GP. Since then, several initiatives have been implemented across the UK founded upon the same principal of targeted service provision for rural communities, frequently using the auction mart as the principal or only base from which to operate. While many of these services continue to operate as mobile services, moving between marts and/or other sites such as agricultural shows, some mart operators have recognized the importance of such service provision and subsequently created purpose-built, permanent clinics within the structure of the mart itself. Several studies have shown that, in the case of mental health, farmers are more likely to seek help if the service provider has some understanding of agriculture [14]. This, along with the increase in the provision of primary health services in rural domains, challenges the stereotyped ‘cultural script’ [39, 40] that male farmers will not seek help for attitudinal reasons, and suggests that other, more practical barriers prevent help-seeking, and that if these barriers are deconstructed, perceived benefits begin to outweigh perceived barriers and therefore allow for new behaviors to form.

The purpose of this article is to explore the role of the livestock auction mart in bringing primary health services to the farmer, and to investigate how the integration of such provisions into both the physical and social structures of the mart might facilitate changes in previously entrenched normative beliefs and behaviors, particularly, but not only, those of men. With one in five farmers in the UK now being women [41], and data emerging that mental health and wellbeing for women has become a ‘substantial concern’ [42] (p.6), women also form a part of this study.

## Method

This analysis stems from empirical data which emerged from a wider study on the social role of auction marts within agricultural communities in the UK. It uses a qualitative approach comprising semi-structured interviews with auction mart stakeholders (farmers, auction mart employees and operators, and individuals representing farmer support organizations).

Interviews were carried out by a member of the research team at 17 locations where marts exist or recently existed at sites across England (*n* = 11), Northern Ireland (*n* = 2), Scotland (*n* = 1) and Wales (*n* = 3), in order to ensure all regions were represented for the wider study of which this research was a part. Where a mart was present, data collection occurred on-site, apart from a small proportion which took place on the telephone. 90 respondents were interviewed in total: 42 farmers, 27 auction operators, 15 individuals from support initiatives (including nurses) and 6 other mart stakeholders (such as chairpersons or trading standards officers). The age of farmers ranged from 25 to 83, and 14% (*n* = 6) of interviewed farmers were women. The majority of interviews were carried out face-to-face. Where interviews were conducted in person, this was carried out in a private space at the auction mart, such as an office. Sampling was purposive, as marts were specifically chosen by the research team. The selection process sought to represent marts from older, smaller and more ‘traditional’ market-formats to newer, out of town, agri-business centers, and those marts occupying an intermediate position. Two sites were chosen specifically because one had a market close in the last ten years and another was reported to be likely to close within the next 12 months. Such a range of marts allowed for the research team to examine whether the presence of a support service might be dependent upon type, size, or location of a mart, as well as allowing a wider exploration of experiences related to marts changing, closing down, or moving, in relation to social isolation experienced by attendees. Marts were also selected according to their geographical location to represent every country within the UK at some level. Further criteria included marts with a demonstrable practice of undertaking functions over and above the sale of livestock, such as hosting primary healthcare services, as well as marts demonstrating more limited functions, or offering none at all. The purpose of this was to determine how perceptions of health service provision at marts might be impacted by such variances in support, among all stakeholders, including those operating the marts. Study sites ranged across private and local authority-owned businesses and different livestock sales types. Once selected, the operator of each mart was invited to participate in the study, and further interviews with farmers and other stakeholders were facilitated by the mart operator or similar stakeholder, usually on market day. Farmer support representatives were approached directly by the research team by phone, email, or on-site at a mart. Again, these individuals were selected purposefully in order to ensure that a variety of ventures were represented, and included national and regional farmer support organizations, as well as staff implementing healthcare services, such as nurses.

Interviews were audio-recorded once written consent was obtained from the respondent, transcribed post-interview, and uploaded and analyzed using the qualitative software package NVivo (V.12.2, QSR International). The analysis process took the form of thematic analysis, loosely following Braun and Clarke’s [43] 6-step process, and was driven by grounded theory [44] due to the field of study being relatively new. Grounded theory is a bottom-up approach to understanding social processes and facilitates the generation of theory from data. Deductive and inductive coding were applied to all texts, populating predetermined themes (such as awareness of, experience of and use of service) and allowing for new themes to emerge (such as role of peer-to-peer learning and the importance of trust in breaking down barriers). Due to the open-ended nature of the health and help-seeking related question ‘Are there any health services provided at your local mart? If so, can you tell me a little bit more about what these are, how useful you think they are, and whether you have ever used them yourself?’, the majority of themes arose inductively, with the primary researcher exploring emerging themes throughout the research process, and the research team being consulted at intervals to review all coding.

Ethical approval for the study was granted by the SSIS Ethics Committee of the College of Social Sciences and International Studies at the University of Exeter, committee reference number 201920–014, and participants were recruited on a voluntary basis. All data were anonymized to help prevent identification of respondents and/or auction mart sites.

## The role of livestock auction marts in promoting help-seeking behaviors

Multiple themes emerged from the qualitative data acquired, which serve to illustrate how livestock auction marts encourage and facilitate help-seeking among farmers, particularly men. Due to interview themes overlapping between the various types of stakeholder interviewed, instead of presenting data separately for each, results are instead presented as themes which blend comments from all interview cohorts. The themes are as follows: perceived barriers to seeking help, impact of delayed help-seeking, the deconstruction of barriers to encourage new behavioral norms, evidence of impact and behavior change, shifts in perceived barriers and benefits, and peer-to-peer learning.

Of the 17 auction mart sites, two hosted permanent clinics, three hosted mobile clinics, four hosted ad hoc services (such as blood pressure checks) and seven revealed that they never hosted any primary healthcare services. The presence of a primary healthcare service services appears not to be dependent upon the size or location of the mart, but upon the individuals and local organisations involved in setting up the service/s. Mobile clinics travelled to several marts a week, although some marts allocate rooms to nurses who then travel between marts. If healthcare practitioners identify a health issue, the protocol is usually to advise that patient to then contact their own doctor, although direct referrals have occurred. Farmers with a pre-diagnosed condition tend to already be managing their condition with their family doctor. These were in the minority among our sample.

Many respondents knew of and/or had experienced health hubs on auction mart sites, but knowledge of such services was not universal, and several auction mart operators were not aware of the presence of primary healthcare services at other marts.

### Perceived barriers to seeking help

According to respondents, the following determinants influence the help-seeking behaviors of people in the livestock farming community, particularly in relation to help from primary healthcare practitioners: attitude/culture, accessibility/convenience, work commitments and time constraints, and faith in primary healthcare provision more generally. None of these are mutually exclusive but some are more physical/structural while others are likely to have developed as a result of experience, indoctrination or other societal influences. All of these determinants were expressed as barriers, cited both by farmers and by other stakeholders within the auction mart community.

#### Attitude/culture

Attitudinal and cultural determinants were reported to play a significant role towards whether a farmer chooses to seek help for an illness or injury. A variety of factors contribute to what might be recognized as hegemonic masculinity, including pride, fear of being judged, or stoicism linked to a more integral identity of ‘being farmer’. Stoicism was referred to more often than any other determinant in terms of barriers to help-seeking.

‘The older generation of farmers, certainly they just keep going and going and going until they drop, and you won’t stop them […] Farming itself is like a disease’ (Farmer 18)

The suggestion was, among some interviewees, that the perceived severity of an illness or injury must be extremely bad before an act of help-seeking occurs.

‘I would persevere for a while […] You just don’t go to the doctor until you really think it is necessary’ (Farmer 26)

Several farmers spoke proudly of the fact that farmers are known for not seeking help. Their language suggested that differences in the perceived severity of an illness or injury imply that farmers who do not seek help for a particular issue were stronger than non-farmers who might be more inclined to seek help for a similar issue.

‘If you go into hospital they ask you what you do, and you say that you are a farmer […] they will admit to you that there must be something wrong with you because farmers don’t go to see about anything *unless there is something wrong*’(Farmer 24)

It was reported by some farmers that they had continued to work for several hours with injuries later diagnosed as broken bones or torn ligaments. Some were reported to have continued to work for days before seeking help.

Several respondents revealed that active behaviors towards help-seeking would be more likely to occur in response to pressure from a female family member, therefore diverting the decision-making process potentially associated with weaker, nonmasculine, feminized behavior away from themselves.

‘You have a wife who tends to book you in, so you have to go’ (Farmer 29)

This push–pull dynamic might be perpetuated in order to maintain ‘masculine capital’ but stoic behavior could also be a result of lack of trust or confidence in the health system. It may also stem from a fear of the unknown or what the implications of having the severity of an illness of injury confirmed might mean for them as individuals, for their family and/or for their business.

‘It is like going to the dentist, going to the doctor. It is the same sort of thing’ (Farmer 35).

Both age and gender were frequently referred to in relation to the expression of stoicism, with older males (typically over the age of 55 years old) considered more likely to delay help-seeking as compared to younger farmers or women in farming. However, two female farmers out of the six interviewed demonstrated similar stoicism to the males regarding seeking help for physical health conditions.

‘[What do you think about the […] nurses?] Never had anything to do with that aspect [Have you not?] No [So if you get sick what do you do?] Usually get right on your own’ (Farmer 6 – female)

‘I’ll only go if I really have to’ (Farmer 25 - female)

Another female farmer compared her attitude to help-seeking to that of her husband and her son, who were also farmers, stating that she and her son would be more likely than the older male in the family to seek help from friends and family if their mental health were to suffer.

‘My husband is one of those. He won’t talk to people, he’s not as good as me and my son at talking to people and saying how he feels’ (Farmer 1 - Female)

Similar attitudes of stoicism related to mental health were displayed by a number of older respondents although several farmers across a range of age groups, as well as auction operators, admitted to having experienced challenges with their mental health. Two respondents openly discussed experiences of depression and the management plans they had in place with their GPs. Seeking help from a practitioner was regarded as a suitable help-seeking route, but many stated a preference to talk to a rural chaplain, friends or family members before a professional. A minority of respondents expressed irritation at the use of terms such as ‘mental health’, dismissing recognition of such issues as weak or overly sensitive. Several older, male farmers expressed discomfort or preferred not to discuss the topic, using dismissive verbal and body language.

#### Loss of faith in service provision

A small proportion of respondents commented on feeling let down by the services that are available to them, either through treatment routes proving to be ineffective, or by the format of appointments being regarded as increasingly unfriendly.

‘He was under the doctors about that, but it didn’t seem to do him any good and I think he’s lost a bit of faith’ (Farmer 1)

A loss of faith, again, became particularly pertinent with regards to mental health treatment. A small number of respondents highlighted that, according to their experience, several individuals were known to have fallen through the gap in terms of receiving appropriate treatment. In such cases, individuals were more likely to seek help from partners, spouses, other family members or the community.

#### Accessibility/convenience

The second most cited determinant likely to impede the ability to seek help from a healthcare professional was accessibility/convenience. Many of the farmers interviewed lived in rural or semi-rural locations and had to travel to local towns or villages in order to see a health professional. Farmers tend to work long hours [9] and choosing to take time out is frequently regarded as detrimental to the business, especially where animals require tending to. In addition, several respondents acknowledged that attendance at a local surgery required the extra effort of changing out of cumbersome, dirty work clothes which proved off-putting to some due to the extra time required to carry out this effort.

‘It’s just time isn’t it […] You need to get changed you can't, you know, you can't go into surgery like this, can you?’ (Farmer 13)

Accessibility emerged frequently as an issue, again attributable either to the rural location of a farmer’s home or infrastructural barriers within a town which might be inaccessible for farming vehicles.

‘If you were to go into town from the auction with trailer or something, it is a nightmare to get to the doctor’ (Farmer 42)

#### Work commitments and time constraints

The average working week for a farmer in the UK is approximately 65 h a week [45], a number which exceeds the average actual weekly hours of work for full-time workers (pre-COVID) which stood at 36.2 h a week [46]. For farmers working with animals, the working day can be further complicated due to milking or feeding times or seasonal commitments such as calving or lambing.

‘As a farmer, you are in a position where you can’t take a day off. There is nobody else to do the job. So, if you are crawling, you have still got to go and feed those animals’ (Farmer 41)

Combined with the more general work obligations of farm work, work commitments and time constraints are high on the list of determinants influencing farmer behavior regarding help seeking.

‘I would go to the doctor, but you have to keep on working, don’t you?’ (Farmer 8)

Several respondents highlighted the likelihood that a livestock farmer would be more likely to attend to the welfare of their animals in place of their own, prioritizing their own health and wellbeing below that of their livestock.

‘These folks will look after their own animals and then neglect themselves’ (Farmer support representative 4)

Respondents frequently stated that the lack of flexibility of available services did not match with the erratic nature of farming, creating general associations between attending in-person appointments and a sense of inconvenience. Several commented on the lack of availability of appointments, particularly in the case of mental health services.

‘When you are depressed, when you are at your weakest, you have to fight the bloody receptionist to get an appointment […] I went to the doctor with suicidal thoughts and they still don’t know whether I am alive or dead’ (Farmer 16)

‘Their only answer is to give you tablets […] They dull everything which is what they did for me, both the highs and the lows, but didn’t deal with the causes at all’ (Farmer 16)

### Impact of delayed help-seeking

By delaying treatment due to any of the above reasons, a number of risks exist. These might be relatively temporary or minor and arise while recovering from an illness or operation. One farmer, for example, delayed seeking help for a hernia and due to the seriousness of the resulting operation, could not work for at least a month post-operation. This potentially impacts on the family, who may be required to work more, or on the farm income as somebody will need to be employed to cover the farm work.

‘He was sort of in self-denial […] He had two grown up sons and a wife and they just couldn’t get him to go and see about this hernia, and it just got so bad’ (Farmer 8)

Potential impacts might, however, be more severe, such as death.

‘A guy just up the road […] he had terrible stomach pains and he had a burst appendix and he died. But he wouldn’t go to the doctor and that was it and then the farm had to be sold’ (Farmer 24)

### Deconstructing barriers to encourage new behavioral norms

The centrality of the mart to agricultural communities clearly remains significant for the many who have, or once traded in, livestock. Market day acts as a fulcrum around which many farmers organize their time and the sites upon which they are held have, as a consequence, been recognized by many as prime locations from which to base primary healthcare and other services.

'A livestock mart is a brilliant way to reach out (Farmer support representative 1)'

Where some format of health hub was present at a mart, the majority of farmers exhibited positive attitudes towards their existence, demonstrated by feedback ranging from acceptance to the active and consistent use of the service/s. One farmer advocated for a health service to be provided at every livestock auction mart.

‘It’s a lot easier to walk through that door than get on the phone, get an appointment with your local doctors to take the morning off' (Farmer 9)

Service professionals attributed this to a number of factors. Although many recognized the inhibiting role of stoicism among potential attendees, they believed that by aiding farmers to bypass the more structural issues, such as inconvenience, time constraints, work commitments, flexibility issues, appointment availability, and developing confidence in the service, stoicism as a determinant was weakened, and no longer acted as a barrier to help-seeking.

‘We’re there, it’s very quick, it’s convenient, there’s nothing mysterious about it, and they seem to like that' (Farmer support representative 2)

The ability to do this was principally attributed to the development of trust.

‘It takes time to build up trust. That’s why so many of these short-term projects fail in a way […] and certainly with the farming community, because it takes time for people to trust you enough. We’ve been so lucky with our nurses because we’ve had wonderful continuity […] Certainly, on the mental health side of things, it takes a lot of courage’ (Farmer support representative 2)

The implication is that trust develops as a result of consistency of service, patience, and time spent building relationships between practitioners and auction stakeholders.

‘Part of the secret of this is the regularity of it, the relationship building. You can’t just waltz into a situation and offer help and expect people to respond. The relationship and the trust is absolutely fundamental’ (Farmer support representative 6)

In many cases, it was also reported that effectual relationships could be built because those implementing the service at the practitioner-farmer interface were individuals who had some knowledge of farming, be it through coming from a farming family or simply being familiar with the agricultural community and the issues with which it is faced.

‘They’re from farming backgrounds but they’re nurses […] Now, how perfect is this?! Somebody who knows what a farmer is like and has the farmer-style conversation. You’ve got to be very, very direct with the farmer’ (Farmer support representative 1)

One farmer support individual, however, refrained from over-emphasizing the importance of a farming background for those running the health services on-site.

‘I think it obviously helps if they have got an understanding of farming [but] as long it’s a, what I call a ‘nice’ person, in the sense that it’s a person you feel comfortable with and you can talk to, then I suppose it doesn’t really matter if they’re farming-related, or not’ (Farmer support representative 15)

For those implementing the service on-site, it was recognized that initial uptake of the service is likely to be slow due to the reticence of farmers to seek help due to all of the reasons outlined above.

‘The hardest thing is getting them across the threshold’ (Farmer support representative 11)

However, as trust is built, perceptions of service provision appear to positively adjust rapidly among mart attendees, usually resulting in not only new perceptions regarding help-seeking itself, but also new behaviors towards seeking help.

### Perceptions of bringing primary healthcare services to the mart

The majority of farmers interviewed viewed the placing of health hubs at auction marts positively, with one farmer having suggested the need for it at their local mart unprompted. Stoicism was still exhibited, but again this might be attributed to expectations to stick to a cultural script within the setting rather than held as an actual belief. Deflecting its relevance to older farmers, for example, might an example of this.

‘I think probably it is a good idea having that health [hub] It is probably more of a question for the older people really’ (Farmer 42)

Those who have experienced some kind of healthcare provision at a mart tend to consider it of benefit, either to themselves or to the community as a whole.

‘I think it’s very good, I mean I’m not afraid because I have been there, I have been there, and I know what goes on’ (Farmer 22)

Those with no experience tended to be more skeptical.

‘Whether you have got bad health or you’ve got a problem of some sort I don’t think the auction is the place for it’ (Farmer 3)

And while only five farmers out of 42 regarded the placement of primary healthcare services within marts as a bad idea, on two occasions this was due to the potential lack of the efficient use of nurses rather than believing the concept itself to be poor.

### Evidence of impact

Due to the strategy of the study, GDPR limitations, differences in record-keeping between service providers, and the fact that a number of relevant stakeholders had not been alerted to the arrival of the researchers, quantitative evidence of direct impact for each study site operating a health hub was not available. However, respondents from each cohort were able to describe the perceived impact of the presence of primary healthcare practitioners on-site. Where a permanent clinic was in place, the footfall was reported by service operators as being consistent, at the very least.

‘We have a drop-in clinic for farmers […] for the bulk of the day which allows farmers to drop in without appointments to have their health worries addressed and they do in considerable numbers' (Auction operator 1)

The only example provided that was described as poorly attended was attributed to the poor timing and short presence of a pop-up hub appearing once on market day.

'So, these people who were trying to do a trial and demonstrate that this is what people want buzzed off [before the sheep were loaded] And course, that immediately skews the statistics […] Well, you don’t just rock up and expect people to use what you’re offering first off […] You gotta stick at it. And I think, if they’d stuck at it, they would have found people' (Farmer Support representative 4)

Numbers of attendees at a health hub appear to vary according to the size of the mart, and how busy a site is on any given sale day. One farmer support organization provided an average figure of 54 people passing through a clinic each market day. Other sites also reported a consistent flow, with between 2 and 17 attendees per day, depending on the type of service offered. The services are always staffed by nurses, although some might also employ other practitioners such as physiotherapists or podiatrists. There was no evidence of any service offering prescription services or direct appointments with GPs on-site.

According to some of the nurses, results from the initial uptake of service provision mirrors results from the study 20 years ago [31, 34] as relatively high numbers of service attendees have significant health issues highlighted.

‘I can certainly think of one case, one auction mart, where the first time it was done, there were almost 30% of the farmers that were seen that were strongly advised that they must see their GP urgently’ (Farmer support representative 6)

The majority of conditions identified tend to be associated with blood pressure, high cholesterol, diabetes, addictions (e.g. alcohol or smoking) or poor dietary choices. These can all be indications of more serious issues. Other issues discussed included musculoskeletal, and in the case of female farmers, continence problems. For all of these issues, attendance at a mart-located primary healthcare service can facilitate a route to improved health.

‘We have had two young people, actually, one of whom smoked very heavily and, as a result of having a health check and getting a bit of a wakeup call, he’s managed to stop smoking. And another guy - young man, who was quite overweight - it shook him into thinking carefully, much more carefully, about his diet and he’s slimmed down and at a much more comfortable weight […] One of our nurses […] she’s picked up countless people who’ve been pre-diabetic’ (Farmer support representative 15)

However, while a constant presence and the build-up of trust was referred to by a number of respondents as being important to encouraging new help-seeking behaviors, even pop-up hubs occurring infrequently were said to have been worthwhile in terms of numbers. This would most likely be what was referred to as a ‘quick MOT’ and involve blood pressure monitoring.

‘She didn’t give me any names, but they took blood pressure of about thirty-five/six people and I think there were about two/three referrals from it as well’ (Auction operator 25)

One farmer revealed how the presence of a pop-up hub on-site encouraged her to accept a vaccination that she would otherwise have been unlikely to pursue independently.

‘This is where I had my flu jab […] Well it saved me, you know, getting changed and going down to surgery [Would you have got it anyway?] I don’t think I would have, no’ (Farmer 13)

In addition to reports of high attendance levels, respondents stated that marts also benefit from the presence of a primary healthcare service due to unexpected health-related events.

'It’s going to be a busy day today. Somebody will collapse out there [Really?] Oh yeah, we know that. That’s why we’ve got a second defibrillator coming. We’ve got one out here. Now we’re getting a second one' (Farmer support representative 14)

Such collapses were reported to stem from heart attacks, diabetes-related incidents or epileptic fits, among other things. Farmer support organization respondents, including nurses, also revealed how crucial a space to discuss issues with a nurse were for the benefit of mental health. It was reported as being a common occurrence that individuals would state that they were seeking help for a physical issue, but that this would develop into a conversation exploring mental health issues. One nurse reported it just as likely that people were seeking help for physical conditions as they were for mental health issues.

Due to the open access nature of the healthcare services provided at most mart sites, meaning that registration with a particular practice or region is not required in order to utilize the service, anybody attending or working at the mart is able to benefit.

‘I went myself three or four months ago […] I did yes, and I was quite shocked. I had high cholesterol and things weren’t maybe what they should be' (Auction operator 21)

One farmer support organization representative suggested that primary healthcare services in their current format are not fit for purpose for rural communities.

‘I think GPs surgeries or health boards […] should be reaching out to rural communities rather than sitting in hospitals and GP surgeries waiting for people to come and see them […] Discriminatory isn’t the right word, but it provides a very lesser service to rural people than it does to other people’ (Farmer support representative 1)

### Shifts in perceived barriers and benefits

Respondents recognized that behavioral norms commonly influencing the likelihood of an individual to attend a GP practice were destabilized by the placement of the service at the mart. While stoic attitudes were demonstrated by farmers at the inception of an initiative, these soon shifted among the majority.

‘They just don’t want to do it [go to the doctor] and that is it. But they are more likely to go to the heath van if it is here, yes’ (Farmer 35)

‘[Do you think if they didn’t go in there they would go and visit their local GP?] Probably not no [Why do you think that might be?] Because they are scared of doctors […] When she [the nurse] first arrived when the market opened, no one really went in there but they did start, and now I think sometimes they come to market just to go in there and see her' (Auction operator 2)

The suggestion is that a fear of doctors or medical practitioners is dissipated by the format offered at the mart.

‘Farmers using [the healthcare service at the mart] will never go to the doctor and get it done and I think it is quite interesting’ (Auction operator 21)

This is likely due to farmers getting to know those providing the service, trust being developed between service providers and the auction community, witnessing and talking to other farmers who have previously used the service, and breaking down barriers through word of mouth.

‘They were doing this health check in the café and they weren’t getting folks to go. Farmers are shy, but once one or two has gone… So, I have talked to a lad next to me and I said come on, I said you go, and I will go, and they will get a few to go then. And so, I went there and I had my health check […] They had a full run of folks all day then going, once they get started. And that is good’ (Farmer 40)

It is also attributable to the convenience provided by the service, breaking down significant structural barriers in place as discussed previously.

‘Farmers are hopeless at going to hospital or the doctors, absolutely hopeless […] Whereas they can come in and drop off their stock at eight o’clock. If they are hanging around to wait for the sale that starts at ten, they generally go for a bit of breakfast and then they can pop back, drop into the clinic, discuss something if they have got a concern and get referred or get it dealt with’ (Auction operator 27)

One respondent suggested that prior to the existence of nurses at the mart, they might have been more likely to go to their doctor, but now rely more on the nurses at the mart than their own general practice doctor.

‘[Why do you think it’s super?] Because you don’t have to book an appointment to see your doctor or a practice nurse [Do you tend to go to the doctor now or do you come here first?] I come here first […] You can more or less see one of these nurses or both of them right away, no waiting for 15 or 20 days or however long it is for an appointment’ (Farmer 5)

There were suggestions among some of the farmer respondents that older farmers might be more inclined to use the services at the mart, but no quantitative evidence exists to support this.

‘Dad does use the health center here […] I probably wouldn’t think of using this one to be fair' (Farmer 2)

‘More for elderly farmers that wouldn’t be going to the doctor’ (Farmer 35)

Some nurses working in health hubs confirm that their patients tend to be older, but they attribute this to the fact that it tends to be the older member of the family who attends the mart, while younger members stay at home on the farm. However, they report that it is not uncommon for younger people to use the service.

Even where health provision exists at a mart, sometimes the stoicism is still too strong to concede to a new behavior, and it cannot be guaranteed that all mart attendees will utilize any primary healthcare services provided.

### Peer-to-peer learning

The effect of peer-to-peer influence appears significant with regards to service utilization, as respondents made it clear that the close-knit nature of the auction mart community facilitated relationships, networks and perceptions of the standing or status of others. One respondent who had never heard of or experienced a healthcare service at a mart expressed negative sentiments towards the concept, due to the potential to be judged if witnessed visiting a nurse by peers.

‘If you went to see the nurse, everybody would be ‘Oooo, he was at the nurse today, what’s wrong with him?’ (Farmer 33)

However, this sentiment was less common than expected and for all respondents who had experienced such a service, perceptions remained extremely positive.

‘It is a nightmare to get to the doctor. You get a doctor’s appointment at whatever time and if you get held up or something changes at the auction and you can’t get there. Whereas if there is someone [at the auction] you can ask if it’s alright if you come back in an hour or whatever. So yeah, I think [a health hub] is probably, is something that would be a good idea’ (Farmer 42)

Several respondents referred to the potential for peer-to-peer influence to help embed service use as part of the everyday mart experience, a potential recognized by auction operators, farmers and farmer support representatives alike.

‘Their mates would be looking interested, and because the nurses record their results on a little take away slip, they would then go and compare notes and their friend would come along and have a check’ (Farmer support representative 2)

‘Because their friends are doing it, everyone else is doing it’ (Farmer 17)

The placement of primary healthcare services at a mart site, among all other recognizable services and activities affiliated with agriculture or rural life, acts to legitimize help-seeking behavior among mart attendees and promote a shift in the ‘cultural script’ currently adhered to by many farmers related to help-seeking [40]. However, one respondent suggested that this might only be a temporary suspension of behavioral norms rather than contributing to more permanent change in belief systems and behaviors, as outside of the mart space, male farmers would fall back on the displacement of decision-making to a female family member.

‘Without the market somebody is going to have to be persuaded by his wife to go and see the doctor’ (Auction operator 25)

## Discussion

Our findings support the premise of the HBM which suggests that optimal behavior change is more likely to be achieved if measures to increase help-seeking behaviors effectively target perceived barriers in particular, but also perceived benefits. They also demonstrate, however, that normative behaviors and beliefs which embody rural masculinity, while gradually transitioning in the wake of campaigns, education, and cultural changes occurring within wider society, are still prevalent in farming and continue to inhibit health-related help-seeking behaviors of farmers. Such behaviors present a risk to individuals at risk of musculoskeletal disorders, work-related injuries, or mental health issues as well as respiratory, skin and diet-related problems. Prolonged delays in health treatment can exacerbate illness and injury and the associated wider impacts stemming from these [7, 11, 14]. By delaying help-seeking for a health or injury issue due to fears that the result of seeking help might negatively impact business, the likelihood is that the impact could be much worse if treatment is delayed. However, our findings also imply that such perceptions regarding hegemonic masculinity and help-seeking might, to some extent, stem from ‘cultural scripts’ [40] rather than being fully representative of the actual situation. This suggests that changes in cultural scripts within farming might be subject to a delay effect, where changes in practice and beliefs, about help-seeking, for example, are likely to occur prior to changes in how these practices and beliefs are communicated to others. Certain perceived barriers around help-seeking can be actively deconstructed through specifically designed, workplace/site-oriented support services, increasing the likelihood of perceived benefits being associated with help-seeking and subtly dispelling the tendency towards stoicism as a reason not to seek help. By placing a primary healthcare service within a space that acts both as an agricultural workplace and as a social hub, by engendering a domain based on trust and familiarity, and by encouraging service use through peer-to-peer demonstration, masculine capital associated with denying or ignoring health conditions can, in some cases, be shifted and beliefs challenged in order to encourage perceptions of help-seeking as a healthy and normalized behavior.

These results suggest that the prevailing reasons behind limited help-seeking behaviors among farmers are physical and structural determinants such as time constraints, work commitments or geographical limitations. The majority of respondent farmers regarded the health hub as a good idea and many reported having used one at least once where they were available. Some even attached pride to the service provided at a local mart and enjoyed telling stories attached to their visits to the nurses. The integration of health hubs within the overall structure of the auction mart proved to be a welcome transition, suggesting that while stoicism and masculine hegemony are the determinants most likely to be cited by most stakeholders operating within agricultural communities, this is in fact due to the ‘script’ rather than being representative of reality. By removing barriers such as time constraints, the need to change out of work clothes, the need to book and travel to an appointment, and thus take time off work, and other more subtle barriers such as mistrust of practitioners, and by bringing the health practitioner to the work-site, in this case the auction mart, stoicism is not sufficiently prohibitive as a determinant to prevent farmers from using the service. Attitudinal and cultural barriers are believed to be broken down through trust and the purpose-specific nature of the services provided, which often employ nurses or other specialists who are knowledgeable about the world of agriculture. The role of the service operator, such as a nurse, is crucial in the deconstruction of these barriers. Trust has always been implicit in the cultural construction of the auction mart, be it of fellow buyers and sellers, or of auction staff [29, 47]. Within the auction mart dynamic ‘in order to be an insider, one must be trusted, and this could only occur by proving possession of the skills and knowledge necessary to keep other insiders safe’ [29], (p. 95). While it is reported not to be imperative that service operators (such as nurses) come from a farming background, the underlying implication is that such a history will facilitate stronger and more trusting relationships within the mart community, leading to greater utilization of service. These service operators act to socially integrate the concept of help-seeking into the everyday networking experienced by the farmer at a mart, as nurses, for example, become known to mart attendees and life-worlds are shared beyond the simple relationship of patient-practitioner. Once a farmer has been persuaded over the threshold, help-seeking behaviors are described as changing rapidly from passive and skeptical to active and accepting. Our findings mirror those of Roy et al. [20] who discovered that previous experience of formal help tended to encourage repeat behaviors, but for those with no experience of receiving formal help, skepticism was more likely.

By facilitating the link between farmers and primary care practitioners, on-site services provided at marts can not only assist with physical illness and injury, but it is often via this route that previously undisclosed mental health issues are unearthed. According to Stark et al. [48], it is common for farmers to present with physical symptoms to their family doctor in the months prior to committing suicide, often without mentioning psychological issues. But service operators suggest that the mart service model might facilitate the divulging of such issues where the normal service model does not, potentially preventing the more severe repercussions of undiagnosed mental health issues.

The service model provided at livestock auction marts acts to reframe help-seeking as a normal part of mart attendance, such as selling animals, buying from the feed store or visiting an on-site hairdresser. By situating a primary healthcare service amidst other modes of consumption, the health hub itself becomes integrated as another form of consumption to be made use of by the consumer. The likely cost–benefit decision process influencing any action towards help-seeking, such as weighing up the need to travel to a local practice with taking time away from the farm, perceived barrier versus perceived benefit [16], might under normal circumstances be made according to the perceived severity of an illness or injury. But the convenience of the mart on site removes the need for this process, stimulating cues to action which might not have occurred outside of the mart setting.

The strength of peer-to-peer influence in farming is shown to support the utilization of healthcare services, and while this is often achieved initially through teasing, cajoling, or by the reluctant approach to the service by farmers, the constant presence of the service quickly becomes normalized and individuals approach the service as they would any other which might be provided at the mart. Studies of ‘masculine capital’ [25, 27] tended to focus on younger respondents, but the case might be that as older farmers are those referenced as using these primary healthcare services at marts the most, such capital becomes less important with age, and that by passing the majority of work to somebody else, such as a son or daughter, some of that capital is also transferred, leaving older farmers more open to being considered ‘less masculine’. At the same time, however, some farmers, especially younger individuals, actively apply health awareness to develop new behaviors. According to de Visser et al. [25], ‘men who resist or reject hegemonic masculinity must develop viable alternative masculine identities’ [25] (p. 1048). They suggest that ‘it may be possible for men to accommodate ‘non-masculine’ behaviors within an overall ‘masculine’ identity’ [25] (1048). Associations of strength in help-seeking might, therefore, be one way of promoting new help-seeking behaviors and just as marts have provided a key space within which to accrue masculine capital, they can equally be used to facilitate shifts in perception towards what constitutes such capital. Of equal importance, by placing the service at the ‘work site’ of the livestock auction mart, it removes the ability to attribute help-seeking behaviors to female members of the family, helping to break down the traditional reinforcement of the gendering of health-related behaviors [10, 11]. In spite of this, primary healthcare provision at livestock markets should be actively promoted to men and women in order to support this aim [27].

Potential issues of service provision at marts include the danger that if a health hub failed, farmers might once again lose trust in the healthcare system. The service provided at the mart might also act to help farmers temporarily suspend their normal perception toward help-seeking, causing them to revert to a previous behavior or belief should it be removed. In addition, as shown by our findings, some farmers have become more reliant on mart healthcare services over and above their usual routes to treatment, which might be detrimental to their overall health due to the limitations of the service provision offered at marts.

### Limitations to the study

The exact number of marts to have participated in such initiatives is unknown. This is due to limited data being available, as well as the gradual closure of many marts across the country potentially preventing such data from being available. Equally, due to the thematic nature of this study, it lacks specific data to quantify findings and determine statistical significance of any of the claims suggested from the findings. A further study to identify exact numbers of services offered by type and frequency of service, with the addition of attendance and impact figures over a particular duration of time, would be an extremely useful trajectory to develop from this research.

This study and the services provided by the healthcare practitioners only deal with a small proportion of the types of health issues faced by farmers, and touches little on workplace injuries. It attempted to examine help-seeking for both physical and mental health issues under one umbrella but would be interesting to explore the role of livestock auction marts on each of these health aspects in more depth.

The majority of studies regarding help-seeking directly examine farmers, subsequently excluding farm workers and other individuals related to working a similar occupation, such as agricultural contractors. The latter often work extremely long hours and further examination of the help-seeking behavior of the workforce as a whole would be beneficial in the adoption of future interventions. At the same time, most academic literature examines help-seeking in terms of male farmers. However, our findings demonstrate that stoicism is not exclusively the domain of the male farmer, therefore more work could be done looking at the help-seeking patterns of female farmers.

Finally, further work would be beneficial to determine how a similar site-based model might be used for other types of farmers unlikely to frequent a livestock auction mart, such as those working in the arable or horticultural sectors.

## Conclusion

Livestock auction marts continue to be entrenched in tradition as well as cultural and behavioral norms. However, they can also act as spaces within which to facilitate the transformation of norms which are outdated and potentially harmful to members of their community. There exists a need to move beliefs from a sense of a reluctance to seek help from being the norm among agricultural communities, removing shame or stigma from the act and instead creating positive associations between choosing to seek help and identity. The creation of positive attitudes towards help-seeking and health service providers is ‘predictive of help-seeking’ [15] (p. 156). The key to encouraging transitions in help-seeking behavior lies not only in focusing on health outcomes but also exposing ‘dominant rural masculinities as a construct to be exposed as inherently unhealthy’ [10] (p. 134). By addressing normative masculinity directly within the domain within which it operates, and using peer-to-peer persuasion and other learning/knowledge exchange/behavior change methods, such norms can gradually be persuaded towards new behaviors.

The health challenges faced by agriculture are multifaceted and require multiple solutions. Whether it is physical or mental health, a first step is to ask for help and support. By situating services within a recognizable, comfortable domain, perceived and actual barriers are removed and, instead, the perceived benefits of help-seeking are nurtured. While consistency and permanence are not essential for a service to be of use, the constant presence of such a service within the lifeworld of the farmer helps to normalize the presence of a health hub and destigmatize associations with attending. This gradual development within livestock auction marts demonstrates that the farmer will not go to the doctor for a multitude of reasons, but if the healthcare professional is brought to the farmer, then fewer barriers remain to their seeking help, potentially impacting not only the farmer, but also their family, their business, and the community within which they function as a whole.

## Data Availability

The datasets generated and/or analysed during the current study are not publicly available due to them containing sensitive material which do not meet the GDPR guidelines but are available from the corresponding author on reasonable request.
